# Isolated tibial deformity is the most prevalent varus pattern in North American patients undergoing medial opening wedge high tibial osteotomy

**DOI:** 10.1002/jeo2.70357

**Published:** 2025-07-24

**Authors:** Takaaki Hiranaka, Takeo Tokura, Nicola D. Mackay, Ryan M. Degen, Kevin R. Willits, Robert B. Litchfield, Alan M. J. Getgood

**Affiliations:** ^1^ Fowler Kennedy Sports Medicine Clinic University of Western Ontario London Ontario Canada; ^2^ Department of Orthopaedic Surgery, Section of Medicine, Division of Medicine, Dentistry and Pharmaceutical Sciences, Graduate School of Medicine, Dentistry and Pharmaceutical Sciences Okayama University Okayama Japan; ^3^ Aspetar Orthopaedic and Sports Medicine Hospital Doha Qatar

**Keywords:** bony deformity, medial opening wedge high tibial osteotomy, total knee arthroplasty, varus alignment

## Abstract

**Purpose:**

To evaluate the location of deformity in varus alignment in a North American population and assess early total knee arthroplasty (TKA) conversion rates and TKA‐free survival following medial opening wedge high tibial osteotomy (MOWHTO) based on the bony deformity location.

**Methods:**

A retrospective analysis was performed on patients with varus alignment who underwent MOWHTO. Deformity analysis measured the hip–knee–ankle (HKA) angle, mechanical medial proximal tibial angle (mMPTA) and mechanical lateral distal femoral angle (mLDFA) using automated software. An abnormal mMPTA was defined as <85° and an abnormal mLDFA was defined as >90°. Cases were classified into four groups based on deformity location: tibial, femoral, combined or no bony deformity. The differences in TKA conversion rates among groups were analysed using the chi‐square test, while TKA‐free survival was determined using Kaplan–Meier survival analysis, with between‐group differences assessed using the log‐rank test.

**Results:**

A total of 271 patients were included (mean age: 51.6 years; mean follow‐up: 3.6 years). The mean HKA angle was 173.0° ± 3.1°. Among the 271 patients, 38% (*n* = 103), 18% (*n* = 48), 11% (*n* = 30) and 33% (*n* = 90) had tibial, femoral, combined and no bony deformity, respectively. TKA conversion rates were 3% (*n* = 3/103), 0% (*n* = 0/48), 7% (*n* = 2/30) and 9% (*n* = 8/90) for the tibial, femoral, combined and no bony deformity groups, respectively, with no significant difference among the groups (*p* = 0.080). Kaplan–Meier survival analysis showed no significant difference in TKA‐free survival among the four groups (*p* = 0.185).

**Conclusion:**

In this North American cohort, various varus deformity locations were analysed, with isolated tibial deformity being the most prevalent. Regardless of deformity location, TKA conversion rates remained low, suggesting that MOWHTO may be beneficial even in patients without isolated tibial deformity.

**Level of Evidence:**

Level III.

**Abbreviations**
BMI: body mass index
CPAK: coronal plane alignment of the knee
DFO: distal femoral osteotomy
DLO: double‐level osteotomy
HKA: hip–knee–ankle
HTO: high tibial osteotomy
JLO: joint line obliquity
K–L: Kellgren–Lawrence
mLDFA: mechanical lateral distal femoral angle
mMPTA: mechanical medial proximal tibial angle
MOWHTO: medial opening wedge high tibial osteotomy
OA: osteoarthritis
TKA: total knee arthroplasty

## INTRODUCTION

Medial opening wedge high tibial osteotomy (MOWHTO) is a widely performed surgical procedure for young, active patients with medial knee osteoarthritis (OA) associated with varus malalignment [5, 31]. While traditionally considered a tibial‐based issue, varus malalignment is now recognised as a multifactorial condition arising from tibial, femoral or combined deformity, as well as intra‐articular degeneration or ligamentous laxity [10, 11, 34]. The location of deformity varies across ethnic groups; for example, Asian populations exhibit predominantly tibial‐based varus malalignment, whereas European populations demonstrate a relatively higher prevalence of femoral‐based deformity [7, 9, 12]. These differences underscore the necessity of tailoring the varus alignment correction strategy to the population being treated.

Recent advances in automated planning tools have significantly improved the ability to identify deformity locations and optimise osteotomy strategies [32, 33]. However, MOWHTO is often performed uniformly, regardless of the underlying deformity. In cases with no tibial bony deformity, this approach may lead to excessive increases in the mechanical medial proximal tibial angle (mMPTA) and joint line obliquity (JLO). Such changes can result in increased shear stress on the articular cartilage, potentially accelerating OA progression and leading to less favourable clinical outcomes [21, 22, 27]. Therefore, it is crucial to analyse the location and presence of bony deformity to optimise osteotomy strategies.

Although the distribution of deformity locations in varus alignment has been studied in certain populations, it remains poorly understood among North American patients. Previous research has rarely focused on the specific locations of deformity in varus knees, and addressing this gap is essential for improving surgical planning and patient outcomes [4, 14]. Accordingly, the primary aim of this study was to retrospectively analyse the location of the deformity among North American patients with varus malalignment who underwent MOWHTO and to simulate osteotomy based on deformity location. The secondary aim was to assess both the conversion rate to total knee arthroplasty (TKA) and TKA‐free survival after MOWHTO, based on the bony deformity location. It was hypothesised that isolated tibial deformity would be the most common deformity type in this North American cohort, and that tibial correction would be the most frequently simulated procedure based on deformity location. Additionally, it was hypothesised that TKA conversion rates and TKA‐free survival would not significantly differ among deformity location groups during short‐term follow‐up.

## MATERIALS AND METHODS

This study was conducted in accordance with the 1964 Helsinki Declaration and its later amendments and approved by the Research Ethics Board of the Western University (REB# R‐24‐236). This study was retrospectively conducted using data from patients who had provided written informed consent at the time of treatment.

All patients who underwent MOWHTO at our institution between January 2018 and July 2022 were retrospectively reviewed. Inclusion criteria were (1) age 18–60 years, (2) an indication for MOWHTO due to medial knee OA, full‐thickness chondral defects, meniscal deficiencies or cruciate and/or collateral ligament deficiency and (3) availability of preoperative full‐leg standing radiographs. Patients were excluded if they (1) had a history of previous osteotomy around the knee, (2) underwent double‐level osteotomy (DLO) or distal femoral osteotomy (DFO), (3) had a follow‐up duration of less than 2 years or (4) lacked appropriate radiographic imaging (e.g., radiographs without a calibration scale or with poor image quality). All surgeries were performed by one of four fellowship trained orthopaedic surgeons. Knee arthroscopy was initially performed to systematically assess the knee, ensuring that the lateral and patellofemoral compartments were well‐preserved. Monoplanar or biplanar osteotomies were performed according to the surgeon's preference. An angular stable plate was placed along the anteromedial aspect of the proximal tibia in all patients. Postoperative rehabilitation followed a standardised protocol. Patients wore a tracker knee brace for 6 weeks. Flat‐foot touch weight‐bearing was allowed immediately after surgery. Weight‐bearing as tolerated was permitted starting at 2 weeks, and full weight‐bearing was allowed from 6 weeks. Crutches were used until a normal, pain‐free gait was achieved. Return to work was generally permitted at 2–3 months, and return to sports was permitted after radiographic confirmation of bone healing, typically after 6 months.

### Radiographic deformity analysis

The Kellgren–Lawrence (K–L) grade of the medial compartment was assessed using anteroposterior weight‐bearing knee radiographs by a single fellowship‐trained orthopaedic surgeon [18]. Postoperative full‐leg standing radiographs were obtained at 6 months following surgery and used for deformity analysis and simulation. Deformity analysis and osteotomy simulation were performed using commercially available software (mediCAD®, Hectec GmbH). All full‐leg standing radiographs were incorporated into mediCAD® for calibration. The software automatically calculated parameters such as the hip–knee–ankle (HKA) angle, mMPTA and mechanical lateral distal femoral angle (mLDFA). Deformity analysis determined the deformity location, with a preoperative abnormal mMPTA defined as <85° (indicating tibial varus) and a preoperative abnormal mLDFA defined as >90° (indicating femoral varus) [7, 25]. The cases were classified into four groups based on the deformity location: tibial, femoral, combined tibial and femoral and no bony deformity. Patients were classified into the ‘no bony deformity’ group if both mMPTA and mLDFA were within normal limits (mMPTA ≥ 85°, mLDFA ≤ 90°), suggesting that the varus alignment was not caused by a structural deformity of the tibia or femur. TKA conversion rates and TKA‐free survival following MOWHTO were analysed across the groups. The cases were regrouped for further comparison of TKA conversion: those with bony deformity (tibial, femoral or combined; bony deformity group) and those without bony deformity (nonbony deformity group).

### Osteotomy simulation

After identification of the primary location of deformity, osteotomy simulation was initiated at the primary deformity site identified in the software, as previously reported [7, 29]. High tibial osteotomy (HTO) was simulated for tibial‐based deformity and DFO was simulated for femoral‐based deformity. Each limb was corrected to achieve a weight‐bearing line ratio of 50%–55%, crossing the lateral tibial plateau spine [35]. To avoid JLO, the postoperative mMPTA upper limit was set at 94°. When a single osteotomy exceeded these limits, a DLO was simulated, with the first osteotomy correcting the primary deformity site to its upper or lower limit, and the second osteotomy completed at the opposite site until the desired correction was achieved. Based on these simulations, the ideal osteotomy options were defined as HTO, DFO or DLO.

### Statistical analysis

Statistical analyses were performed using IBM SPSS Statistics for Windows, version 29.0 (IBM Corp.). Continuous variables are presented as mean ± standard deviation, while categorical variables are expressed as the count and percentage. The Mann–Whitney *U*‐test was applied for continuous variables, and the chi‐square test was used for categorical variables. TKA‐free survival was analysed using Kaplan–Meier survival analysis and compared between groups using the log‐rank test. Differences were considered significant at *p* < 0.05. The intraclass correlation coefficient with the 95% confidence interval was used to evaluate interrater and test–retest reliabilities, with an intraclass correlation coefficient >0.75 indicating excellent agreement [20]. Two independent examiners assessed the HKA angle in a blinded manner to evaluate interrater reliability, while radiographic variables were remeasured 2 weeks after the initial assessment to evaluate test–retest reliability. These assessments were performed using radiographic data from a randomly selected subset of 20 patients. The overall interrater and test–retest reliabilities of the HKA angle were 0.992 and 0.997, respectively, demonstrating excellent consistency.

## RESULTS

**Figure 1:**
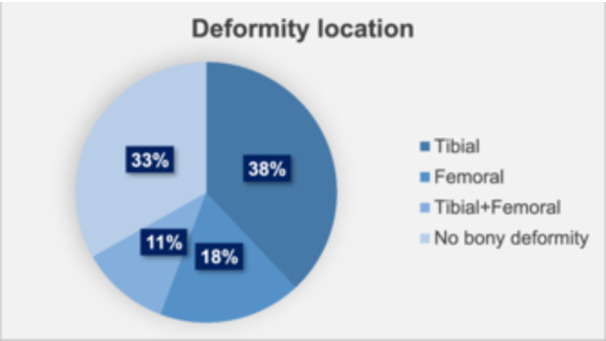
Analysis of varus deformity locations showing that 38% of knees had tibial deformity, 18% had femoral deformity, 11% had combined deformity and 33% had no identifiable bony deformity.

A total of 341 patients underwent MOWHTO at our institution between January 2018 and July 2022. Of these, 70 patients were excluded due to previous osteotomy procedure around the knee (*n* = 5) inadequate preoperative full‐leg standing radiographs (*n* = 65). Consequently, 271 patients were included in the final analysis. The mean patient age was 51.6 ± 8.4 years (range: 21–60 years), with 176 males and 95 females. The mean body mass index (BMI) was 31.1 ± 5.7 kg/m². The mean follow‐up period was 3.6 ± 1.0 years (range: 2.0–6.0 years). The preoperative K–L grade distribution was as follows: K–L grade 1 in 11 knees, grade 2 in 140 knees, grade 3 in 94 knees and grade 4 in 26 knees. The mean HKA angle was 173.0° ± 3.1° in varus (range: 178.0°–164.5°). Among the 271 cases analysed, 67% (*n* = 181) exhibited bony deformity, 38% (*n* = 103) had tibial deformity, 18% (*n* = 48) had femoral deformity and 11% (*n* = 30) had combined deformity. In contrast, 33% (*n* = 90) had no identifiable bony deformity (Figure 1).

**Figure 2:**
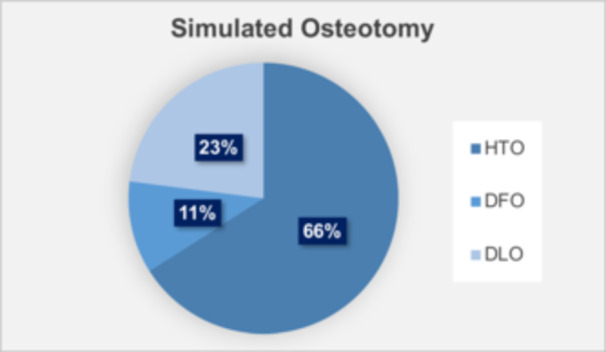
Distribution of ideal osteotomy levels based on simulation data. HTO was recommended for 66% of knees, DFO for 11% and DLO for 23%. DFO, distal femoral osteotomy; DLO, double‐level osteotomy; HTO, high tibial osteotomy.

MOWHTO was performed primarily for OA (87%, *n* = 237), followed by medial meniscus repair (5%, *n* = 13), ligament reconstruction (5%, *n* = 14) and cartilage repair (3%, *n* = 7). Ligament reconstruction procedures included reconstruction of the anterior cruciate ligament, posterior cruciate ligament and posterolateral corner, performed either individually or in combination. Figure 2 illustrates the distribution of ideal osteotomy levels based on simulation data, which indicated that HTO was necessary for 66% of the cases (*n* = 179), DFO for 11% (*n* = 31) and DLO for 23% (*n* = 61) to achieve the a priori determined correction.

Patient demographics, radiographic parameters and simulated osteotomy levels by deformity location are shown in Table 1. There was no significant difference in the reason for MOWHTO (medial knee OA, medial meniscus repair, ligament reconstruction or cartilage repair), age or K–L grade between the deformity location groups. The combined deformity group had the largest varus alignment (mean HKA angle: 168.9 ± 2.5°), whereas the no bony deformity group had the smallest varus alignment (mean HKA angle: 174.8 ± 2.4°) (*p* < 0.001). Simulated osteotomy predominantly involved HTO for tibial (92%) and no bony deformity (86%), DFO for femoral deformity (65%) and DLO for combined deformity (87%).

**Table 1:** Patient demographics, radiographic parameters and simulated osteotomy levels by deformity location.

		Deformity location				*p* value
		Tibial	Femoral	Combined (tibial + femoral)	No bony deformity	
Indications for osteotomy (OA/MM repair/ligament reconstruction/cartilage repair)		92/6/2/3	42/1/2/3	26/0/4/0	77/6/6/1	0.136
Age		52.6 ± 8.3	49.4 ± 9.8	52.2 ± 7.6	50.3 ± 8.9	0.111
Preoperative K–L grade (1/2/3/4)		4/53/32/14	3/20/20/5	0/15/13/2	4/52/29/5	0.452
Preoperative HKA angle (°)		172.6 ± 2.5	172.9 ± 2.4	168.9 ± 2.5	174.8 ± 2.4	<0.05*
Simulated osteotomy type (%)	HTO	92% (*n* = 95/103)	6% (*n* = 3/48)	13% (*n* = 4/30)	86% (*n* = 77/90)	<0.05**
	DFO	0% (*n* = 0/103)	65% (*n* = 31/48)	0% (*n* = 0/30)	0% (*n* = 0/98)	N.A.
	DLO	8% (*n* = 8/103)	29% (*n* = 14/48)	87% (*n* = 26/30)	14% (*n* = 13/90)	<0.05***

*Note*: Values are presented as mean ± standard deviation.

Abbreviations: DFO, distal femoral osteotomy; DLO, double‐level osteotomy; HKA, hip–knee–ankle; HTO, high tibial osteotomy; K–L, Kellgren–Lawrence; MM, medial meniscus; N.A., not applicable; OA, osteoarthritis.

* Statistically significant (*p* < 0.05) in all comparisons except between the tibial and femoral groups.

** Statistically significant (*p* < 0.05) in all comparisons except between the tibial and no bony deformity groups and between the femoral and combined groups.

*** Statistically significant (*p* < 0.05) in all comparisons except between the tibial and no bony deformity groups, and between the femoral and no bony deformity groups.

**Figure 3:**
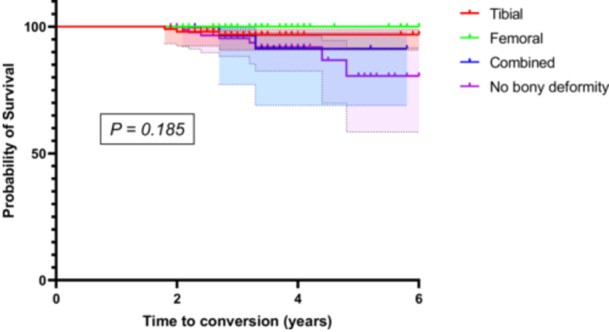
Kaplan–Meier survival curve for TKA conversion among deformity locations. The probability of survival (absence of TKA conversion) is plotted against time (years) for each group: tibial deformity (red), femoral deformity (green), combined deformity (blue) and no bony deformity (purple). No statistically significant difference was observed among the groups (*p* = 0.185). TKA, total knee arthroplasty.

Conversion to TKA occurred at different rates among the deformity groups: 3% (*n* = 3/103), 0%, 7% (*n* = 2/30) and 9% (*n* = 8/90) in the tibial, femoral, combined and nonbony deformity groups, respectively (Table 2). However, no significant difference in conversion rates was observed among the four groups (*p* = 0.080). Kaplan–Meier survival analysis demonstrated no statistically significant difference in time to TKA conversion among the four groups (tibial, femoral, combined and no bony deformity) over the follow‐up period (*p* = 0.185; Figure 3). In addition, TKA conversion rates were compared based on the indication for MOWHTO (medial knee OA, medial meniscus repair, ligament reconstruction and cartilage repair), but no significant difference was observed (*p* = 0.750). Kaplan–Meier survival analysis also showed no statistically significant difference in TKA‐free survival across these groups (*p* = 0.701).

**Table 2:** TKA conversion rates and times after MOWHTO among the deformity groups.

Deformity location	Number of cases (*n*)	Conversion to TKA (*n*)	Conversion rate (%)	Time to conversion (years)
Tibial	103	3	3	2.2 ± 0.4
Femoral	48	0	0	N.A.
Combined (tibial + femoral)	30	2	7	3.0 ± 0.3
No bony deformity	90	8	9	3.2 ± 0.9

*Note*: Values are presented as mean ± standard deviation unless otherwise indicated.

Abbreviations: MOWHTO, medial opening wedge high tibial osteotomy; N.A., not applicable; TKA, total knee arthroplasty.

**Figure 4:**
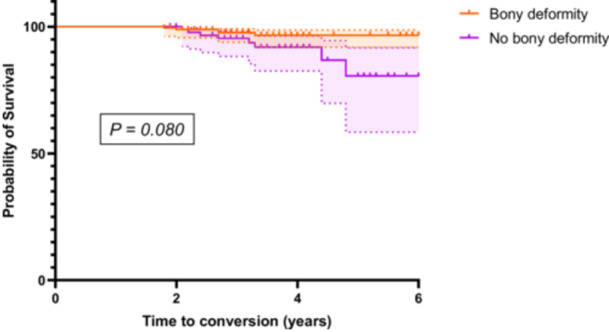
Kaplan–Meier survival curve for TKA conversion in patients with and without bony deformity. The probability of survival (absence of TKA conversion) is plotted against time (years) for each group: with bony deformity (orange) and without bony deformity (purple). No statistically significant difference was observed between the two groups (*p* = 0.080). TKA, total knee arthroplasty.

Combining those cases into those with and without a bony deformity, there was a 3% TKA conversion rate (*n* = 5/181) in those with a preoperative bony deformity versus a 9% TKA conversion rate (*n* = 8/90) in those without a bony deformity who underwent MOWHTO (*p* = 0.068). Kaplan–Meier survival analysis demonstrated no statistically significant difference in time to TKA conversion between two groups (*p* = 0.080; Figure 4).

In the nonbony deformity group, eight knees were eventually converted to TKA. Among these, four knees (50%) had simulated osteotomy levels for HTO, whereas the remaining four (50%) had simulated osteotomy levels for DLO. Conversely, of the 82 knees from the nonbony deformity group that did not convert to TKA, 74 (90%) were simulated for HTO and 8 (10%) for DLO. The proportion of DLO simulations was significantly higher in knees that were ultimately converted to TKA (50%) than in those that were not (10%) (*p* = 0.010).

Preoperative demographic and radiographic data were compared between the TKA‐converted and nonconverted groups. No statistically significant differences were observed in age (*p* = 0.192), sex (*p* = 0.340), BMI (*p* = 0.199), K–L grade (*p* = 0.776), location of the bony deformity (*p* = 0.080), preoperative HKA angle (*p* = 0.338), preoperative mMPTA (*p* = 0.784), preoperative mLDFA (*p* = 0.551) and preoperative joint line convergence angle (*p* = 0.106).

## DISCUSSION

This study revealed that, in a North American cohort, various varus deformity locations were observed, with isolated tibial deformity being the most prevalent. Additionally, two‐thirds of cases exhibited bony deformity, while one‐third had no identifiable bony deformity. Regardless of the specific deformity location, the conversion rate to TKA remained low, suggesting that even patients without isolated tibial deformity may still experience a beneficial treatment effect from MOWHTO. Further long‐term follow‐up studies are necessary to optimise treatment strategies for different varus phenotypes.

To our knowledge, this is the first study to analyse the deformity location in patients undergoing MOWHTO for varus knees within a North American population. Deformity distribution varies geographically [13]. In Japan, tibial and no bony deformity have been reported as the most common types [1], while a German study found a higher prevalence of no bony deformity [7]. An Italian study of TKA patients similarly demonstrated varied patterns of deformity [34]. In our North American cohort, tibial, femoral, combined and no bony deformity accounted for 38%, 18%, 11% and 33% of cases, respectively, illustrating notable regional differences. The substantial proportion of patients without bony deformity (33%–45% across regions) suggests that varus alignment is not always associated with clear structural abnormalities in the tibia or femur. This emphasises the need to carefully assess the underlying causes of varus alignment.

In our cohort, osteotomy simulation indicated that 66% of cases required HTO, 11% required DFO and 23% required DLO. Previous studies have reported notable differences in simulated osteotomy strategies. For example, a Japanese study applying stricter angular thresholds reported a higher rate of HTO [1], while a French study using ESSKA criteria identified nearly half of cases as not requiring osteotomy [26]. A German study with a 2° valgus correction target found more frequent use of DLO [7]. These differences likely reflect variations in alignment targets and cutoff values for mLDFA, mMPTA and joint line orientation across regions. In our study, applying an mMPTA upper limit of 94° led to two‐thirds of cases requiring HTO and one‐third requiring DFO or DLO. Such variability highlights the challenges in comparing outcomes across studies and underscores the need for further validation of simulation‐based planning.

Several factors have been associated with the conversion to TKA following MOWHTO, including age [16, 17, 23], BMI [8, 23], preoperative OA severity [8, 16, 23] and sex [17, 30]. For example, age ≥ 65 years and BMI > 30 kg/m² have been linked to significantly lower long‐term survival rates after HTO [8, 16]. Preoperative K–L grade >2 has also been shown to increase the risk of early conversion to TKA [21], and female patients have been reported to have a higher risk compared to males [23, 30]. In the present study, no significant differences in age, sex, BMI, K–L grade or deformity location were observed between patients who underwent TKA and those who did not. Notably, deformity location was not associated with TKA conversion, suggesting that MOWHTO may be effective regardless of the specific location of bony deformity.

In this study, TKA conversion rates and TKA‐free survival did not differ significantly among the four deformity groups (*p* = 0.080 and *p* = 0.185, respectively). These findings are consistent with a recent report showing no significant differences in clinical outcomes after MOWHTO among various preoperative coronal plane alignment of the knee categories, including tibial‐driven, femoral‐driven and neutral alignment types [36]. Together, these results suggest that postoperative outcomes following MOWHTO may be independent of deformity location or the presence of bony deformity. Given the comparable TKA‐free survival across groups, MOWHTO remains a viable joint‐preserving option for patients with different varus alignment patterns.

The optimal treatment strategy for patients without bony deformity remains unclear, as the benefits of MOWHTO in this subgroup have not been fully established. Although no significant differences in TKA conversion rate (*p* = 0.068) or TKA‐free survival (*p* = 0.080) were observed, the small number of conversion events may have limited statistical power and increased the risk of a type II error. One possible explanation for the slightly higher conversion rate in this group is the difficulty in avoiding excessive JLO or mMPTA, which may negatively impact outcomes [2, 19, 29]. Prior studies have shown that DLO can mitigate such alignment issues [3, 15], and simulated DLO was more common in knees that later underwent TKA (50%) than in those that did not (10%), suggesting its potential role. These simulation results may offer additional context for surgical planning, particularly in patients without clear tibial or femoral bony deformity.

Importantly, arthroplasty outcomes after HTO have been shown to be comparable to primary TKA [6, 24, 28], and the conversion rate in our nonbony deformity group remained low at 9%. Therefore, MOWHTO appears to be a viable option even for patients without clear bony deformity. Further long‐term studies are warranted to refine selection criteria and optimise treatment for various varus phenotypes.

This study has some limitations. First, as a retrospective analysis, variations in surgical decision‐making among the surgeons may have influenced the choice of isolated HTO. Second, while TKA conversion rates were evaluated, some patients who underwent TKA at external medical facilities may not have been accounted for due to the lack of a unified healthcare record system. Moreover, the absence of postoperative patient‐reported outcomes and radiographs limite the comprehensiveness of clinical assessments. In addition, the inclusion of younger patients (aged 18 years and older) may affect the interpretation of TKA conversion rates, as these individuals are less likely to require arthroplasty within a short follow‐up period. Lastly, the relatively short follow‐up period (mean 3.6 years, minimum 2 years) is a limitation of this study and may not fully reflect the long‐term survivorship of MOWHTO or capture late conversions to TKA. Future studies with longer follow‐up durations and comprehensive radiographic and patient‐reported outcome data are necessary to validate and expand upon our findings.

## CONCLUSIONS

In this North American cohort, various locations of varus deformity were analysed, with isolated tibial deformity being the most prevalent. Regardless of the specific deformity location, the conversion rate to TKA remained low, suggesting that even patients without isolated tibial deformity may still experience a beneficial treatment effect from MOWHTO.

## AUTHOR CONTRIBUTIONS

Alan M. J. Getgood and Takaaki Hiranaka designed the study. Takaaki Hiranaka, Takeo Tokura and Nicola D. Mackay analysed and interpreted the data. All authors contributed to data collection and interpretation, and critically reviewed the manuscript. All authors have read and approved the final version of the manuscript.

## CONFLICT OF INTEREST STATEMENT

The authors declare no conflicts of interest.

## ETHICS STATEMENT

This retrospective study was conducted in accordance with institutional guidelines and the Declaration of Helsinki and was approved by the institutional review board of the hospital. Written informed consent was obtained from all patients.

## Data Availability

The data that support the findings of this study are available from the corresponding author upon reasonable request.
